# Gray level co-occurrence matrix and wavelet analyses reveal discrete changes in proximal tubule cell nuclei after mild acute kidney injury

**DOI:** 10.1038/s41598-023-31205-7

**Published:** 2023-03-10

**Authors:** Igor Pantic, Jelena Cumic, Stefan Dugalic, Georg A. Petroianu, Peter R. Corridon

**Affiliations:** 1 Faculty of Medicine, Department of Medical Physiology, Laboratory for Cellular Physiology, University of Belgrade, Visegradska 26/II, 11129 Belgrade, Serbia; 2 University of Haifa, 199 Abba Hushi Blvd, Mount Carmel, 3498838 Haifa, Israel; 3 Department of Pharmacology, College of Medicine and Health Sciences, Khalifa University of Science and Technology, PO Box 127788, Abu Dhabi, UAE; 4 Faculty of Medicine, University of Belgrade, University Clinical Center of Serbia, Dr. Koste Todorovica 8, 11129 Belgrade, Serbia; 5 Department of Immunology and Physiology, College of Medicine and Health Sciences, Khalifa University of Science and Technology, PO Box 127788, Abu Dhabi, UAE; 6 Healthcare Engineering Innovation Center, Biomedical Engineering, Khalifa University of Science and Technology, PO Box 127788, Abu Dhabi, UAE; 7 Center for Biotechnology, Khalifa University of Science and Technology, PO Box 127788, Abu Dhabi, UAE; 8 Indiana Center for Biological Microscopy, Indiana University School of Medicine, Indianapolis, IN USA

**Keywords:** Imaging, Computational biology and bioinformatics, Biomarkers, Health care, Nephrology

## Abstract

Acute kidney injury (AKI) relates to an abrupt reduction in renal function resulting from numerous conditions. Morbidity, mortality, and treatment costs related to AKI are relatively high. This condition is strongly associated with damage to proximal tubule cells (PTCs), generating distinct patterns of transcriptional and epigenetic alterations that result in structural changes in the nuclei of this epithelium. To this date, AKI-related nuclear chromatin redistribution in PTCs is poorly understood, and it is unclear whether changes in PTC chromatin patterns can be detected using conventional microscopy during mild AKI, which can progress to more debilitating forms of injury. In recent years, gray level co-occurrence matrix (GLCM) analysis and discrete wavelet transform (DWT) have emerged as potentially valuable methods for identifying discrete structural changes in nuclear chromatin architecture that are not visible during the conventional histopathological exam. Here we present findings indicating that GLCM and DWT methods can be successfully used in nephrology to detect subtle nuclear morphological alterations associated with mild tissue injury demonstrated in rodents by inducing a mild form of AKI through ischemia–reperfusion injury. Our results show that mild ischemic AKI is associated with the reduction of local textural homogeneity of PTC nuclei quantified by GLCM and the increase of nuclear structural heterogeneity indirectly assessed with DWT energy coefficients. This rodent model allowed us to show that mild ischemic AKI is associated with the significant reduction of textural homogeneity of PTC nuclei, indirectly assessed by GLCM indicators and DWT energy coefficients.

## Introduction

Acute kidney injury (AKI) denotes an abrupt and rapid reduction in the kidney’s ability to perform excretory functions and other roles that support homeostasis^1^. There are numerous causes of acute kidney injury, such as blood hypoperfusion, dehydration, sepsis, effects of various toxins, as well as urinary tract obstructions. These conditions can, in turn, be classified as pre-renal, intrinsic, and post-renal forms of injury. Many AKI symptoms and signs are nonspecific and include confusion, drowsiness, nausea, diarrhea, dehydration, and decreased urine output. Also, some AKIs do not present with obvious symptoms and are very difficult to diagnose. Morbidity and mortality due to AKI are relatively high, and so is the cost of treatment. According to some data, AKI remains one of the most significant financial burdens on healthcare systems, considering the number of hospitalizations and the funding needed for each hospital stay^2,3^.

It is well known that AKI is strongly associated with damage to the proximal tubule and that the dysfunction or death of proximal tubule cells (PTCs) is often the main consequence of AKI^4^. Cells within this epithelium often undergo programmed cell death or necrosis depending on the severity of the injury, while in some very mild AKI cases, the PTC dysfunction is barely noticeable and reversible without intervention^5^. Distinct patterns of transcriptional and epigenetic alterations often follow the dysfunction of PTCs^6–8^. Even during the early stages of AKI, the expression of numerous genes is increased or decreased, which may or may not result in structural changes in PTC nuclei. To this date, AKI-related nuclear chromatin redistribution in PTCs is poorly understood. It is unclear whether changes in PTC chromatin patterns can be detected using conventional microscopy approaches during mild AKI. Also, it is unknown if structural changes in PTC nuclei in mild or moderate AKI have any diagnostic or research value in pathology and nephrology.

In microscopy, various ways exist to evaluate and quantify structural changes in cell nuclei. In recent years, two contemporary and innovative computational techniques have emerged as potentially valuable methods for identifying discrete morphological changes in nuclear chromatin architecture that are not visible during the conventional histopathological exam. The first technique is based on gray level co-occurrence matrix (GLCM) analysis, and it is commonly used to quantify nuclear textural features such as uniformity, homogeneity, and entropy^9,10^. The second technique is based on the discrete wavelet transform (DWT), a mathematical approach to the texture frequently applied for two-dimensional signal analysis as an addition to GLCM. Previous research has shown that both methods are potentially valuable tools in pathology for differentiating damaged and intact cells^11,12^. Also, both methods can be used to train and develop artificial intelligence (AI) machine learning models, such as those based on decision trees, logistic regression, or artificial neural networks^13–17^.

The objective of our study was to investigate if it is possible to apply GLCM and DWT computational methods in the evaluation of pathologically altered proximal tubule cells following acute ischemia–reperfusion injury (IRI)s. We also aimed to propose hypothetical machine learning models that would use GLCM and DWT data as inputs and could predict PTC damage with relatively high classification accuracy. Finally, our objective was also to compare the accuracy and discriminatory power of machine learning models with subjective microscopic evaluation of PTCs and to provide insight into the scientific value of computational methods in this area of pathology.

In this work, we present findings indicating that GLCM and DWT methods can be successfully used in nephrology to detect subtle morphological alterations of PTC nuclear chromatin associated with mild tissue injury demonstrated in rodents. Our results show that mild ischemic AKI is associated with the reduction of textural homogeneity of PTC nuclei quantified by GLCM and the increase of nuclear structural heterogeneity indirectly assessed with DWT energy coefficients. To the best of our knowledge, this is the first study to quantify GLCM and DWT indicators of PTC nuclear structure after AKI and the first to show that these indicators can be used to separate cells from damaged and intact tissue. In this article, we also propose creating random forest and support vector machine algorithms that theoretically have relatively high accuracy and discriminatory power in classifying PTC nuclear structural patterns associated with AKI. Such AI-based solutions might, in the future, pave the way for digital pathology-based platforms that support enhanced quantitative histopathologic assessments that allow us to explore and extract information beyond human visual perception^18^.

## Results

### Serum creatinine, blood urea nitrogen, and histological analyses

**Figure 1:**
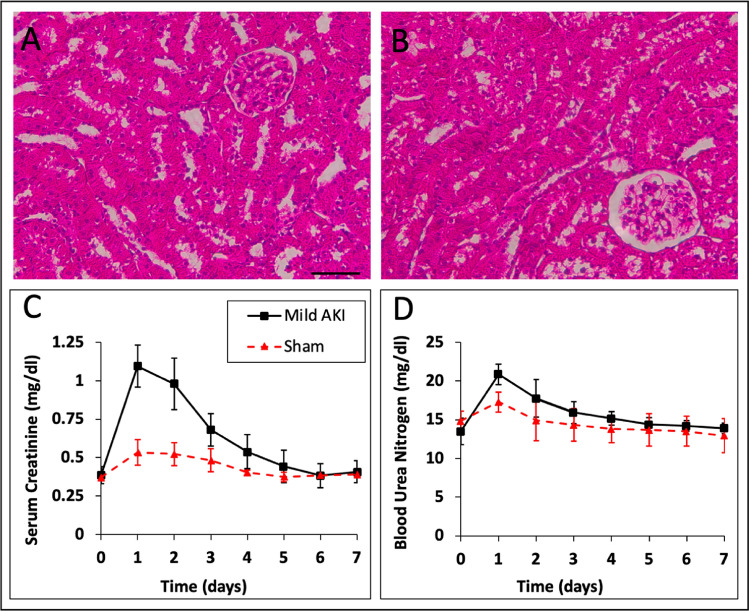
Histological and serum creatinine analyses conducted on rats in the sham and mild AKI groups. Brightfield microscopic images highlight the mild disruptions to tubular and glomerular integrities observed in kidney sections obtained from rats with (**A**) sham and (**B**) mild AKI. Similarly, the plots in (**C**, **D**) reveal the transient and substantial elevations in serum creatinine and blood urea nitrogen levels associated with a mild form of IRI, compared to the sham groups, respectively.

We monitored serum creatinine (SCr) and blood urea nitrogen (BUN) levels in both groups of animals. The levels observed among the control (sham) rats showed minor and non-significant (p = 0.068) and (p = 0.058) fluctuations in SCr and BUN concentrations from the 0-h to the 24-h mark post-procedure. Moreover, these values remained within normal baseline levels (0.3 ≤ SCr ≤ 0.6 mg/dl) and (10.5 ≤ BUN ≤ 18.8 mg/dl) compared to the measurements obtained from animals with mild IRI. In contrast, the creatinine and blood urea nitrogen levels in the acutely injured animals rose abruptly and significantly (p < 0.01) and (p < 0.01) above their original ranges at the 0-h mark during the first 24 h after injury (0.9 ≤ SCr ≤ 1.2 mg/dl and 19.4 ≤ BUN ≤ 22.3). These levels gradually returned to baseline within the 7-day measurement period (Fig. 1C,D). both serum creatinine and blood urea nitrogen measurements differed significantly across the 7 days (p < 0.05) and (p < 0.05) respectively from the levels measured in sham animals. Likewise, brightfield images collected from cortical sections in the sham group revealed subtle alterations to the renal microarchitecture (Fig. 1A), and more pronounced disruptions were observed after inducing the mild form of IRI (Fig. 1B). Altogether, these changes to normal creatinine levels and the renal ultrastructure correlate with standard biochemical and histological findings^19–25^.

### GLCM analysis

**Figure 2:**
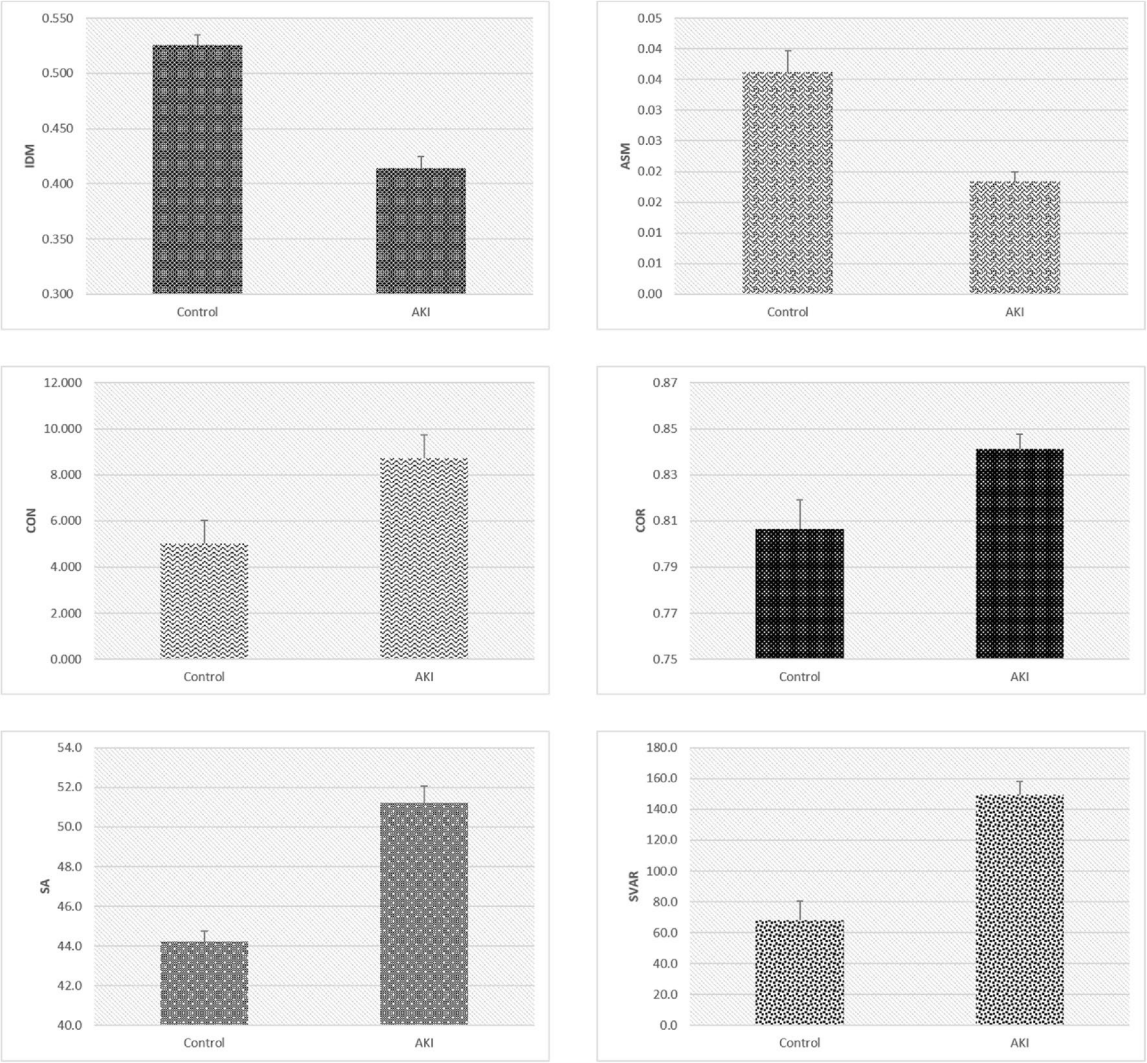
GLCM indicators used to distinguish the two groups. Mean values and standard deviations of nuclear GLCM indicators in mild AKI and control groups.

The average values of the angular second moment and inverse difference moment in the controls were 0.036 ± 0.003 and 0.53 ± 0.01, respectively. In contrast, in the AKI group, the values were decreased and equaled 0.018 ± 0.001 and 0.41 ± 0.01, respectively (Fig. 2). Differences between AKI and controls were statistically highly significant for both GLCM indicators (p < 0.01). This result implied that both local textural homogeneity and uniformity of PTC nuclei decrease due to AKI. Conversely, the mean values of textural contrast increased after AKI from 5.02 ± 0.52 (controls) to 8.73 ± 0.66 (p < 0.01), and a similar, although less pronounced increase was observed in the GLCM correlation feature (0.806 ± 0.013 to 0.841 ± 0.006, p < 0.01). The mean values of textural Sum Average and textural Sum Variance indicators also increased, and the change was highly significant (p < 0.01). In the control group, these values equaled 44.21 ± 0.53 and 68.36 ± 11.98, respectively, while in the AKI group, they equaled 51.21 ± 0.86 and 149.47 ± 8.36, respectively.

**Figure 3:**
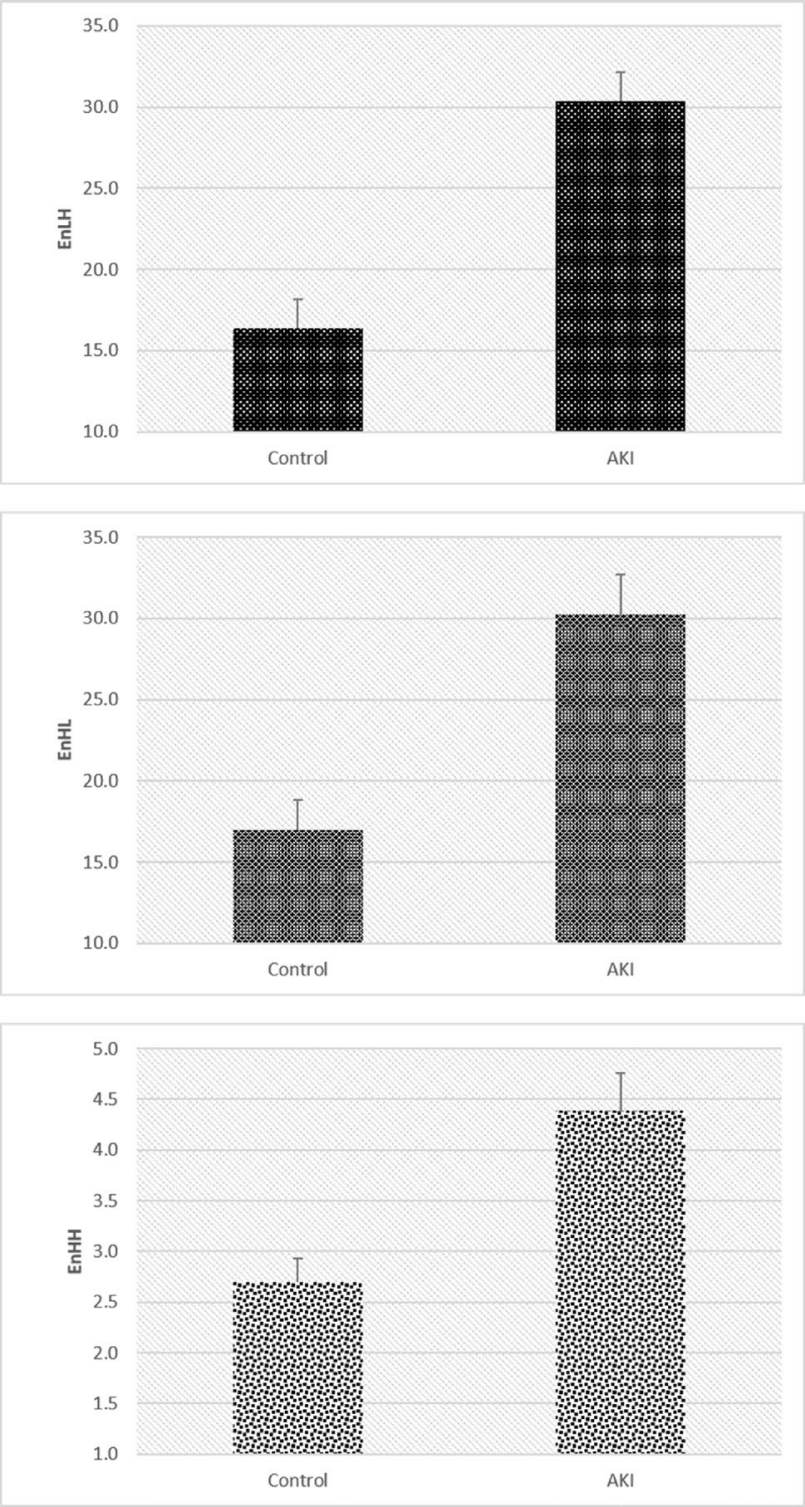
Discrete wavelet transform coefficient energies used to distinguish the two groups. Mean values of discrete wavelet transform coefficient energies of PTC nuclei in mild AKI and control groups.

The mean values of discrete wavelet transform coefficient energies increased in AKI, correlating with the previous GLCM results showing the reduction of textural homogeneity and uniformity. The wavelet coefficient energy, EnLH, obtained with the use of a combination of low-pass and high-pass filleters showed an increase from 16.38 ± 1.80 in the controls to 30.37 ± 1.75 in AKI (p < 0.01), while a similar increase was observed for its EnHL counterpart (16.98 ± 1.83 to 30.27 ± 2.39), (p < 0.01). The wavelet coefficient energy, EnHH, in the control group equaled 2.69 ± 0.23; in the AKI group, it equaled 4.39 ± 0.37 (p < 0.01). Figure 3 shows the mean values and standard deviations for DWT indicators.

**Figure 4:**
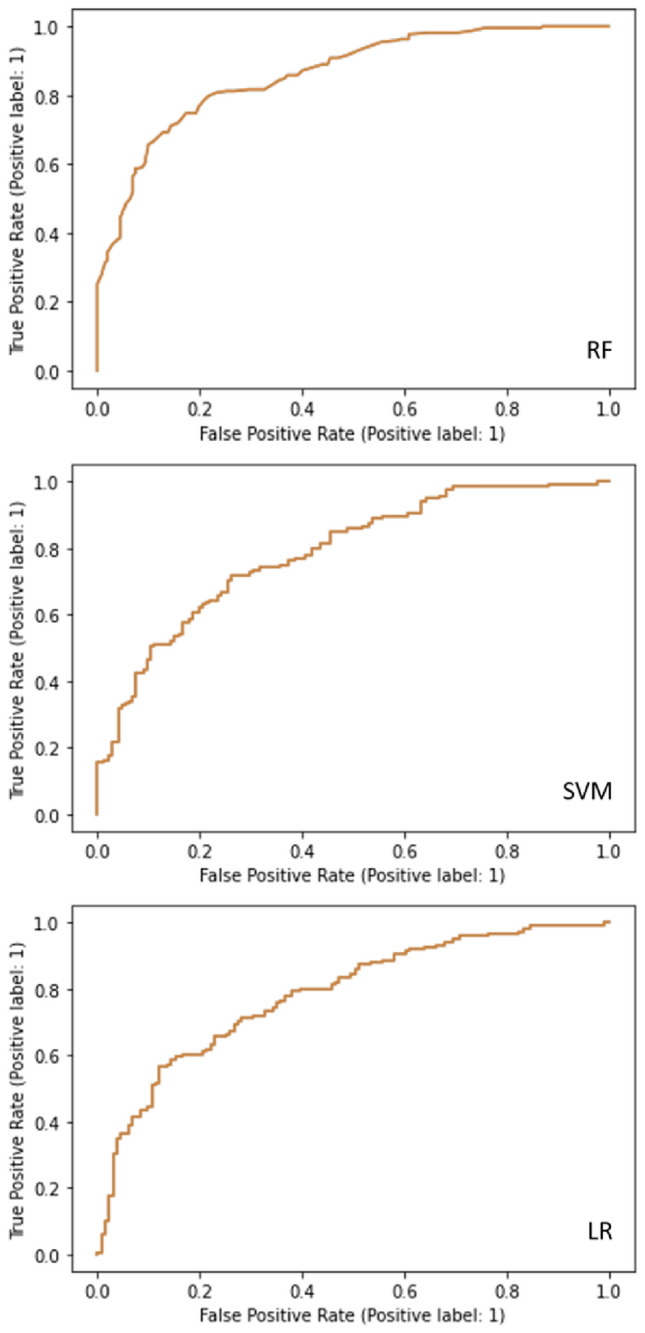
Receiver operating characteristic curves. Receiver operating characteristic curves for hypothetical random forest (RF), support vector machine (SVM), and binomial logistic regression^26^ machine learning models.

In this work, we propose creating three hypothetical machine learning models based on logistic regression^26^, support vector machine (SVM), and random forest (RF). Of the three trained and tested models, the highest accuracy, equalling 0.79 was determined for the random forest algorithm. The model had excellent discriminatory power in terms of potential ROI classification, and the area under the receiver operating characteristics curve equaled 0.86. The ROC curves for each model are presented in Fig. 4. The ssupport vector machine model had an estimated accuracy of 0.73 and the area under the ROC curve of 0.79. The logistic regression model presented the lowest accuracy of 0.69 with the area under the ROC curve of 0.78. These results indicate that the random forest classifier has the greatest potential in future development of computer-aided sensing systems for the identification of acute kidney injury.

Results obtained from the subjective evaluation of PTC nuclear ROIs indicated that the classification accuracy of this approach was only 55.1%. In the sample of AKI nuclear ROIs, only 10.7% were correctly identified to belong to the AKI class (true positives). The ROC analysis showed that the area under the curve was 0.55 indicating poor discriminatory power. These results demonstrated the inability of the conventional microscopic assessment to detect changes in PTC nuclei associated with AKI.

## Discussion

Even though AKI is highly variable in its clinical presentation, damage to proximal tubule epithelium is a significant hallmark of the condition that can manifest through various mechanisms that elicit pre-renal, intrinsic, and post-renal injuries. For instance, the degree of injury, clinical severity, and progression of AKI are associated with the duration of IRI. The current understanding of AKI has been developed through extensive research using animal models, particularly mouse and rat species, and the ability to rapidly and reliably induce mild, moderate, and severe damage in the PTCs by administering nephrotoxins in various doses^27^ and modulating the duration needed to induce IRI^28^. Based on these facts, in this work, we demonstrate the ability of gray level co-occurrence matrix and discrete wavelet transform methods to detect subtle structural changes in PTC nuclei after mild pre-renal AKI.

Serum creatinine and blood urea nitrogen are conventional functional biomarkers of AKI^29–33^. These peak serum creatinine and blood urea nitrogen elevations 24 h post-IRI and the gradual return to baseline within a week are hallmarks of this model19. Furthermore, we observed greater than a 1.5-fold rise in SCr (approximately 2) but less than a 20:1 ratio (approximately 19:1) for BUN:SCr indicating an injury that is just on the border of progressing to a more severe condition^34^. Examining images collected from sham and mildly injured animals showed that the most critical nuclear GLCM indicators, such as angular second moment and inverse difference moment, significantly change as the result of mild AKI, which indicates the rise of nuclear textural. To our knowledge, this is the first study to combine GLCM indicators and DWT coefficient energies to reveal discrete AKI-related alterations in PTC nuclear architecture. We also propose hypothetical machine learning models based on support vector machines, random forest, and logistic regression, which might, in the future, be used as a part of accurate computational AI sensing systems for diagnostic purposes.

Probably the most important aspect of our study is that we demonstrated that computational methods are superior in detecting AKI-related discrete alterations in PTC nuclei compared to subjective microscopic evaluation. In general, the histological changes after AKI are often relatively small, even when physiological and biochemical indicators of kidney function indicate severe damage. Such subtitles were previously demonstrated on numerous occasions, and some authors even state that this is one of the reasons why many acute tubular necrosis cases are not appropriately diagnosed. For example, Ho and Morgan (2022) state that “renal histological changes in AKI are disproportionally mild compared to the corresponding reduction in glomerular filtration rate” and that this is a potential problem in nephropathology protocols.

The subjective assessment of nuclear chromatin patterns revealed that even an experienced professional in chromatin microscopy could not successfully determine if the PTC belonged to the AKI or control group. Phenomena associated with the nuclear injury, such as condensation and marginalization of chromatin, were not visible nor identifiable using conventional means. As mentioned in the results, only 10.7% of the nuclear ROIs were correctly identified as belonging to the AKI class (true positives), which could perform better when this subjective evaluation is regarded as a statistical model. The resulting classification accuracy, under the ROC curve of less than 60%, confirms poor discriminatory power in separating AKI from non-AKI PTCs. On the other hand, even the poorest performing machine learning model based on a relatively simple logistic regression approach presented an accuracy of 0.69 with the area under the ROC curve of 0.78, which is a considerably better performance.

The random forest model is the most suitable for the future development of advanced AI-based sensing systems for detecting damaged kidney cells. This characteristic is probably due to the specific methodological characteristics of this ensemble learning method, where multiple decision trees are constructed during training, reducing the chance of overfitting. In the future, this model would have to be trained and validated on a much larger sample and in a different setting where a much greater number of experimental animals is used. After that, one may foresee creating a simple, affordable, and user-friendly computer application that could be used as an addition to the conventional pathology assessment of biopsy samples.

In our previous articles, we applied similar GLCM approaches to analyze alterations to the renal vascular architecture^35^, highlighting its potential application in the characterization of whole organ scaffolds19 that can be generated for bioartificial kidney development^36^. Using this technique, we also examined cell nuclei after the damage induced by exposure to a sublethal toxic dose of ethanol^12^. We calculated angular second moment, inverse difference moment, textural contrast, GLCM correlation, and variance on an experimental model of saccharomyces cerevisiae, and we demonstrated that these features significantly change after alcohol treatment. This type of cell damage was also associated with reducing textural homogeneity and uniformity, leading us to believe that these changes in nuclear textural patterns are generally related to cell damage. Similarly, as in the present study, we proposed several machine learning models, such as the ones based on logistic regression, decision trees, and artificial neural networks^12^. Despite the apparent differences in the methodological approach and experimental protocol between the two works, this assumption is worthy of investigation in future research. The random forest model is the most suitable for the future development of advanced AI-based sensing systems for detecting damaged kidney cells. This characteristic is probably due to the specific methodological characteristics of this ensemble learning method, where multiple decision trees are constructed during training, reducing the chance of overfitting. In the future, this model would have to be trained and validated on a much larger sample and in a different setting where a much greater number of experimental animals is used. After that, one may foresee creating a simple, affordable, and user-friendly computer application that could be used as an addition to the conventional pathology assessment of biopsy samples.

One of the earliest research articles on applying GLCM in the histological evaluation of kidney tissue was published in 2013. Indicators such as the angular second moment and inverse difference moment were quantified to assess chromatin architecture in macula densa cells during mice postnatal development and aging^37,38^. Textural features were evaluated in conjunction with fractal dimension and lacunarity as indicators of complexity. Although no statistically significant changes were detected in ASM and IDM values, the research nevertheless holds some value since it was the first to show that GLCM analysis is not only possible in kidney tissue but also applicable for evaluating structural alteration in cell nuclei. The results showed that nuclear patterns of kidney cells after conventional histological staining could be used to obtain high-quality GLCM data for quantifying nuclear textural uniformity and homogeneity.

Our current study is also one of many to use the GLCM computational method to detect AKI. Previously, textural features such as GLCM contrast and GLCM correlation were quantified in the kidney medulla after inducing IRI in rats by clamping both renal vascular pedicles and subsequent reperfusion with saline^39^. It was shown that both CON and COR features had excellent discriminatory power in separating injured from control medullar tissue, with the area under the receiver operating characteristic curve in both cases higher than 85%. The value of the study is reflected in the fact that the high performance of the method was achieved without the need to train machine learning models. The results identified fractal and GLCM parameters as suitable candidates for developing computational biosensors in nephropathology.

Several potential explanations for the AKI-related changes in PTC nuclear textural patterns were detected in our current study. First, it is possible that AKI led to the redistribution of euchromatin and heterochromatin in PTCs, and that the redistribution resulted from either direct damage to the cell or activation of a signaling pathway. AKI is associated with sometimes profound epigenetic changes, as explained in detail by other authors^6^. Some upregulated genes during AKI may influence chromatin integrity and remodeling on higher scales. Although these phenomena are generally not noticeable during the standard histopathological evaluation, the subsequent changes in textural patterns may have been detected with GLCM and DWT. Also, it should be considered that, sometimes, euchromatin and heterochromatin, at least in the ultrastructural sense, have different levels of fractal complexity^40^, and these differences in complexity may have reflected on GLCM and DWT features in this experimental setting as well. Unfortunately, it is still unclear to which extent the fractality of nuclear structure influences textural GLCM and wavelet indicators of chromatin distribution.

Another possibility is that mild AKI in the renal cortex is sometimes associated with programmed cell death. Indeed, PTCs are highly susceptible to apoptosis, as discussed earlier^41^, and this type of cell death contributes to the loss of kidney functionality during AKI. On the other hand, some previous works have suggested that GLCM indicators such as angular second moment and inverse difference moment significantly decrease after cell treatment with proapoptotic substances^42^. During the early stages of programmed cell death, phenomena such as (initial) condensation and marginalization of chromatin may perhaps lead to increased textural heterogeneity, detectable using both GLCM and DWT. Changed euchromatin/heterochromatin ratio associated with nuclear damage,often not visible during conventional microscopic analysis, may also affect textural indicators. However, additional research is needed to confirm this assumption, particularly on PTCs and other cell populations in the renal cortex.

In the future, it could be possible to broaden this type of research by developing AI models based on artificial neural networks. This approach could include relatively simple perceptron networks, complex neural networks with Bayesian inference, and convolutional neural networks (CNNs). The input layer of neurons in these models could receive DWT and GLCM data, but also the data from a three-dimensional matrix of values based on red, green, and blue light intensities. Including various other image analysis quantifications, such as fractal dimension, lacunarity, and granularity, might further benefit the network’s ability to distinguish damaged from intact cells. Convolutional neural networks are of particular interest since they have been successfully applied for image classification on numerous occasions^43,44^. Creating a complex CNN that combines DWT and GLCM with other input parameters could lead to the development of a sensitive, accurate, and affordable computer-aided diagnostic system that might be an essential addition to current nephropathology practices.

Our study had several significant limitations that need to be discussed and considered when conducting future research in this scientific area. First, there needs to be more literature data on the quality assurance and validity of GLCM and DWT methods in nephropatology and nephrohistology research. Different software platforms often produce different results, and based on our previous experience, indicators such as angular second moment and inverse difference moment can significantly vary depending on the software settings and parameters during micrograph acquisition. Second, one must stress that the AI models proposed in this research are only hypothetical since they were trained and tested on a minimal number of nuclear ROIs. To increase validity and test this approach's diagnostic value, one would need to develop the machine learning models on an extensive sample of micrographs with one ROI corresponding to one individual micrograph or even one individual animal., Also, from our previous experience, values obtained through GLCM and DWT analyses greatly depend on the histological staining applied to the tissue. In the future, one might consider repeating the experiments and using other techniques such as periodic acid–Schiff, Sirius Red, Feulgen, or Toluidine Blue. Only then would we have complete insight into the actual scientific value of GLCM and DWT computational methods.

Finally, a significant limitation of the study is related to the difficulty of connecting the observed changes in nuclear GLCM and DWT indicators to any physiological or pathological phenomenon. This difficulty arises from the fact that GLCM and DWT methods are relatively new in terms of their applications in cell biology, so it is still being determined how exactly processes such as apoptosis and necrosis reflect on nuclear features. As previously mentioned, it is believed that nuclear injury leads to increased textural heterogeneity, manifesting through the reduction of GLCM features such as angular second moment and inverse difference moment. However, the biological mechanisms behind these changes remain unexplored.

In conclusion, we present evidence that GLCM and DWT computational methods can detect subtle structural alterations in PTC nuclei associated with AKI. After quantifying textural features such as the angular second moment and inverse difference moment of nuclear architecture, we conclude that this form of injury leads to the rise of nuclear textural heterogeneity. This change needs to be clearly visible during a conventional histopathological evaluation. Since this syndrome rarely has a sole and distinct and is frequent among patients without critical illness, it is essential that healthcare professionals, especially those without specialization in renal disorders, detect it easily^45^. Thus, we propose creating AI models that use GLCM and DWT indicators as input data, capable of AKI identification and PTC classification with accuracy and discriminatory power much more remarkable when compared to the subjective evaluation of nuclear patterns. The obtained results present a valuable foundation for future research in AI applications in pathology, nephrology, and related disciplines and support current regimens used to address AKI.

## Materials and methods

### Mild acute kidney injury

The study was conducted in accordance with the Indiana University School of Medicine Institutional Animal Care and Use Committee, the Animal Research Oversight Committee at Khalifa University of Science and Technology, and the ARRIVE guidelines. All procedures were approved by these organizations and carried out using these relevant regulations and guidelines on 200 to 400 gm male Sprague Dawley rats (Harlan Laboratories, Indianapolis, IN, USA) to ensure that animals were treated ethically and humanely. The animals were anesthetized using 5% isoflurane delivered in oxygen (Webster Veterinary Supply, Devens, MA, USA) and then given intraperitoneal injections of 50 mg/kg of pentobarbital (Hospira, Inc., Lake Forest, IL, USA). Each rat was then placed on a heating pad to maintain normal physiological temperature, and intraperitoneal incisions were made to expose both renal pedicles for the injured and sham groups. Bilateral micro-serrefine clamps with delicate, atraumatic serrations (Fine Science Tools, Foster City, CA, USA) were applied to occlude blood flow for 15–20 min (animals in the sham group was not subjected to the bilateral clamps). After this period, the clamps were removed to reinstate renal blood flow, and the animals were allowed to recover fully post ischemia–reperfusion and sham injuries. It should also be noted that, throughout our studies, the rats were given free access to standard rat chow and water.

### Serum creatinine and blood urea nitrogen measurements

Blood samples were collected from the injured and sham rats daily across a week in 1 mL Eppendorf heparin-treated tubes after making small incisions on their tails. These samples were centrifuged at 100,000–130,000 rpm for 10 min. The supernatants were then stored at 4 °C. A quantitative determination of creatine kinase activity in serum was then estimated with Pointe Scientific CK (Liquid) Reagents (Point Scientific, Inc., Canton, MI, USA). Measurements were performed with a Beckman Creatinine Analyzer 2 (Beckman Instruments, Brea, CA, USA) according to the manufacturer’s specifications and reported values in milligrams per deciliter (mg/dL). Approximately 10 μL of each serum sample was added to the working reagent (1000 μL), and the absorbance was immediately measured using a microplate reader. Likewise, blood urea nitrogen levels were investigated with the Liquid Urea Nitrogen (BUN) Reagent Set (Pointe Scientific, Canton, MI, USA) according to the manufacturer’s specifications. Briefly, a 1000 μL working reagent was prepared by 1 part of the coenzyme combined with 5 parts of the enzyme reagent, to which 10 μL of each serum sample was added, and absorbance was immediately measured using a microplate reader.

### Histology

Euthanasia was performed on day 7 to obtain whole kidneys from the two groups of animals. For this process, the animals were again anesthetized with pentobarbital, and once fully sedated, whole kidneys were acquired after each renal pedicle was clamped. Once removed, the kidneys were fixed with 4% paraformaldehyde for 24 h at 4 °C. The samples were then immersed in 10% neutral buffered formalin or 4% phosphate-buffered formalin, again for a minimum of 24 h at room temperature. The specimens were then rinsed in distilled H_2_O and stored in 70% ethanol. For infiltration, the specimens were dehydrated through a graded series of ethanol (70%; 80%, 95%, and 100%; two changes each under vacuum for 45 min at room temperature). The specimens were cleared in two changes of xylene (under vacuum at room temperature for 45 min each), infiltrated with 4 changes of paraffin (under vacuum at 59 °C; 45 min each), and embedded in fresh paraffin. After which, 4–5 μm thick sections were cut using a Reichert-Jung 820 microtome (Depew, NY, USA). The sections were flattened on a warm water bath and mounted on coated and charged glass slides. After drying, the sections were deparaffinized, rehydrated, and stained with hematoxylin and eosin (H&E). Images were then acquired with a Nikon Microphot SA Upright Microscope equipped with a sensitive Diagnostic Instruments SPOT RT Slider color camera (Nikon, Tokyo, Japan) and a 20 × objective.

### Computational analysis

**Figure 5:**
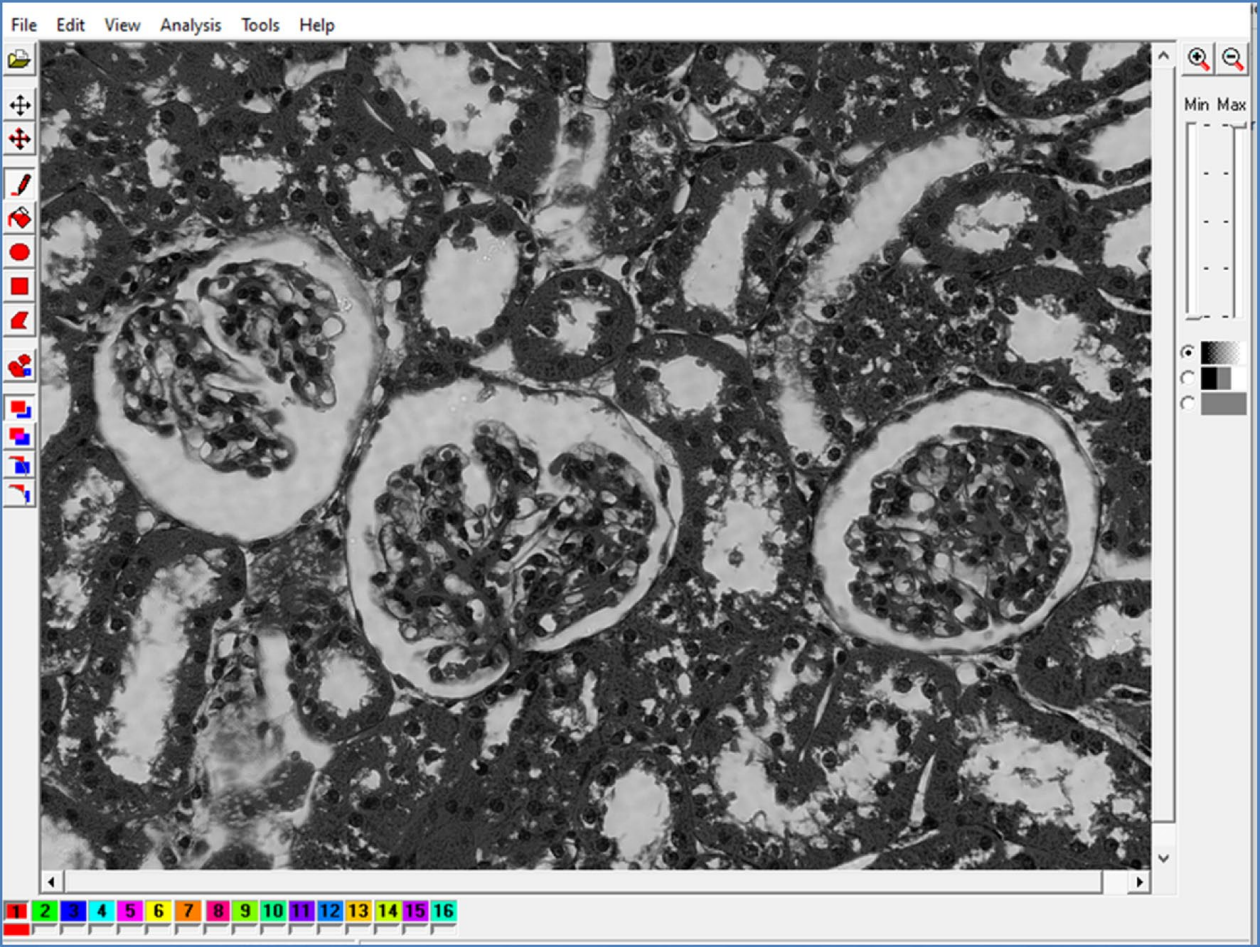
Example of Mazda user interface with a digital micrograph of kidney cortex with AKI. The micrographs were converted to grayscale BMP format for ROI creation and subsequent GLCM and DWT analyses.

We created digital micrographs of the renal cortex with dimensions of 1600 (width) × 1200 (height) resolution units in uncompressed TIF format and RGB photometric interpretation. The micrographs had both horizontal and vertical resolution of 96 dpi and a bit depth of 48. They were later converted to BMP format with the same dimensions and a bit depth of 24 and analysed in a grayscale setup (Fig. 5). For GLCM and DWT analyses we used Mazda software previously developed for the needs of COST B11 European project "Quantitative Analysis of Magnetic Resonance Image Texture" (1998–2002) and COST B21 European project "Physiological modelling of MR Image formation"^46–49^. A similar micrograph analysis protocol was used in the previous publications^11^. Briefly, a total of 1400 regions of interest of PTC nuclei were analyzed: 700 from animals with mild AKI (100 per animal) and another 700 from control animals. For each ROI, 6 GLCM indicators were quantified: angular second moment^50^, inverse difference moment (IDM), GLCM contrast (CON), GLCM correlation (COR), GLCM sum average (SA), and textural sum of variance (SVAR).

The angular second moment is a textural feature that quantifies the uniformity of the two-dimensional signal and can be calculated from the probability (p) values for changes between gray levels i and j:

$$\text{ASM} = \sum_{\text{i}}\sum_{\text{j}}{\left\{{\text{p}}\left(\text{i, j}\right)\right\}}^{2}$$

Here, the resolution units are assigned values taking into account their gray level intensities. Another similar feature that quantifies textural local homogeneity of the signal is inverse difference moment, which is determined as:

$$\text{IDM }\text{=}\sum_{\text{i}}\sum_{\text{j}}\frac{1}{{\text{1} + \left(\text{i-j}\right)}^{2}}\text{ p(i,j)}$$

In this work, we also quantified textural correlation (gray-level linear dependency) and textural contrast (local intensity variation) as additional features which supplement angular second moment and inverse difference moment:

$$\mathrm{CON}=\sum_{\mathrm{i}}\sum_{\mathrm{j}}{\left(\mathrm{i}-\mathrm{j}\right)}^{\mathrm{k}}{\mathrm{P}}_{\mathrm{d}}{\left[\mathrm{i},\mathrm{j}\right]}^{\mathrm{n}}$$

$$\mathrm{COR}=\frac{\sum_{\mathrm{i}}\sum_{\mathrm{j}}\left(\mathrm{ij}\right)\mathrm{p}\left(\mathrm{i},\mathrm{ j}\right)- {\upmu }_{\mathrm{x}}{\upmu }_{\mathrm{y}}}{{\upsigma }_{\mathrm{x}}{\upsigma }_{\mathrm{y}}}$$

where in the normalized GLCM, μ is the mean of rows x and y, and σ the standard deviation^51–53^.

Sum average of the GLCM as the mean of the gray level sum distribution of the image and Sum variance as the indicator of gray level textural dispersion around the mean^51^ were quantified as:

$$\mathrm{SVAR}=\sum_{ }{\mathrm{ip}}_{\mathrm{x}+\mathrm{y}}(\mathrm{i})$$

$$\mathrm{SVAR}={\sum }_{ }{\left[\mathrm{i}-\sum_{ }{\mathrm{ip}}_{\mathrm{x}+\mathrm{y}}(\mathrm{i})\right]}^{2}$$

In addition to the quantification of GLCM textural features, we also applied discrete wavelet transform analysis^46^ by performing linear transformation of data vectors with the vectors having a length of an integer power of two. The vectors were transformed into numerical vectors that had the same length. A special filtering cascade was applied after the separations of rows and columns of data which included the use of high (H) and low-pass (L) filters. We quantified 3 DWT wavelet coefficients (d) energies for the specific combination of filters for the first subband at successive scales: EnLH, EnHL, and EnHH. Considering the subband location (x, y), the scale, and the ROI number of resolution units (n), the energies can be determined as:

$$\mathrm{E}=\frac{{\sum }_{\mathrm{x},\mathrm{y}\in \mathrm{ROI}}{({\mathrm{d}}_{\mathrm{x},\mathrm{y}}^{\mathrm{subband}})}^{2}}{\mathrm{n}}$$

In this study, we also proposed the creation of 3 hypothetical AI machine learning models: support vector machine (SVM), random forest (RF), and a model based on the binomial logistic regression^26^. The models were created in scikit-learn open source, commercially usable machine learning library for the Python programming language^54^. The models were trained using the GLCM and wavelet indicators as inputs on 80% of the ROIs while the remaining data were used for testing. We determined the average accuracy and discriminatory power (area under the receiver operating curve) in terms of the ability of the model to assign the ROI to the AKI or the control kidney tissue. The data were analyzed using Pandas, an open source data analysis and manipulation Python library, as well as IBM SPSS 25.0 statistical analysis software. The Mann–Whitney *U* test was used to determine whether the increases in serum creatinine observed across the first 24 h after injury was significant. All variables are expressed as mean ± standard deviation, and for all evaluations, a *p* value of less than 0.05 was considered statistically significant.

### Subjective evaluation and classification of the PTC nuclei

As an addition to this study, we performed subjective evaluation and classification of PTC nuclei. The aim of the evaluation was to assess the classification accuracy and the related area under the ROC curve of a subjective observer in terms of the ability to blindly assign an AKI or non-AKI class to a nucleus based on its morphological appearance. The aim was also to compare this classification accuracy with the accuracy of SVM and RF machine learning models. The analysis was done blindly by a microscopy expert (IP) with previous experience in morphological evaluation of altered chromatin architecture in physiological and pathological conditions. The observer was presented with a total of 1400 PTC nuclear ROIs (700 belonging to AKI and 700 belonging to the control group) from the micrographs described above. The ROIs were selected in ImageJ software (National Institutes of Health, Bethesda, MD) and the surrounding area of the micrograph was cleared by selecting the “Clear Outside” option (ImageJ > Edit > Clear Outside). The observer was then asked to assign each ROI with the value “1” for AKI class or “0” for the non-AKI (control) class based on his subjective opinion on the morphological characteristics of a nucleus. Particular attention was given to the structural patterns indicating chromatin condensation, chromatin marginalization, early karyolysis or karyorrhexis, or any alterations of nuclear size or shape. After the subjective evaluation was finished, the “true” class for each nuclear ROI were revealed. Finally, based on these subjective and “true” classes, classification accuracy of the observer was estimated ROC and analysis was performed to determine the discriminatory power.

## Data Availability

The datasets generated during and/or analyzed during the current study are available from the corresponding author on reasonable requests.
